# Changes from 1986 to 2018 in the prevalence of obesity and overweight, metabolic control and treatment in children with type 1 diabetes mellitus in a Mediterranean area of Southeast Spain

**DOI:** 10.1186/s12887-022-03330-1

**Published:** 2022-05-12

**Authors:** María Teresa Pastor-Fajardo, María Teresa Fajardo-Giménez, Vicente María Bosch-Giménez, José Pastor-Rosado

**Affiliations:** 1 Department of Pediatrics, Hospital General Universitario de Elche, Elche, Spain; 2 Department of Clinical Analysis, Hospital General Universitario de Elche, Elche, Spain; 3 Department of Pharmacology, Pediatrics and Organic Chemistry, University of Murcia, Murcia, Spain

**Keywords:** Obesity, Overweight, Type 1 diabetes mellitus, Childhood, Adolescence

## Abstract

**Background:**

In recent decades, a global increase in the prevalence of childhood overweight and obesity has been observed in children and adolescents with type 1 diabetes.

**Methods:**

This retrospective, cross-sectional, population study examined three groups (1986, 2007, and 2018) of children and adolescents aged < 16 years diagnosed with type 1 diabetes. Overweight and obesity were defined according to the World Health Organization recommendations.

**Results:**

The prevalence of overweight and obesity in diabetic children and adolescents was 30.2% (95% CI: 23.1–38.3). There was a significant increase from 1986 to 2007 (11.9% to 41.7%, *p* = 0.002) and from 1986 to 2018 (11.9% to 34.8%, *p* = 0.012), but no significant differences were found from 2007 to 2018 (41.7% to 34.8%, *p* = 0.492). The age at diagnosis was lower in the group with excess body mass (*p* = 0.037). No significant differences were observed in age (*p* = 0.690), duration of diabetes (*p* = 0.163), distribution according to sex (*p* = 0.452), metabolic control (HbA1c, *p* = 0.909), or insulin units kg/day (*p* = 0.566), between diabetic patients with overweight or obesity and those with normal weight. From 2007 to 2018, the use of insulin analogs (*p* = 0.009) and a higher number of insulin doses (*p* = 0.007) increased significantly, with no increase in the prevalence of overweight and obesity.

**Conclusions:**

The prevalence of overweight and obesity in diabetic children and adolescents increased in the 1990s and the beginning of the twenty-first century, with stabilization in the last decade. Metabolic control and DM1 treatment showed no association with this trend.

## Background

A global increase in the prevalence of childhood overweight and obesity has been noted in the last few decades [1], with a tendency toward stabilization in recent population studies [2, 3]. Similarly, the incidence and prevalence of type 1 diabetes (DM1) has increased in the pediatric population [4–6], and excess weight has become increasingly common in diabetic children and adolescents [7]. The coexistence of overweight and obesity with DM1 should be considered both as a causal factor in the rise in incidence and as a consequence of the disease and its treatment [8]. The accelerator hypothesis postulates that insulin resistance associated with obesity accelerates the DM1 process [9]. Obesity, by promoting inflammation and autoimmunity, could lead to beta-cell failure, but the relationship is not as clear as that between obesity, increased peripheral tissue insulin resistance, and the development of type 2 diabetes [10]. A higher prevalence of overweight and obesity has been associated with intensive treatment [11], influenced by other factors such as lower educational level, onset during puberty, and sedentary lifestyle [12]. However, the increased prevalence of obesity in some diabetic patients may simply mirror an increase in obesity in the population [13].

In our study, we evaluated three groups (1986, 2007, and 2018) of children and adolescents with DM1. The objectives were: (a) to determine the variation over the last 30 years in the prevalence of overweight and obesity in these patients, according to international childhood growth criteria [14]; and (b) to observe possible associations between nutritional status and sociodemographic factors such as age, sex, and obesogenic environment, as well as clinical factors including duration of diabetes, metabolic control, and insulin therapy.

## Methods

### Design, setting, and study subjects

This retrospective cross-sectional population study comprised three cohorts (1986, 2007, and 2018) of all children and adolescents under 16 years of age followed in the Pediatric Endocrinology Unit of a tertiary hospital and diagnosed with DM1 according to the recommendations of the *International Society for Pediatric and Adolescent Diabetes* [15]. The 1986 and 2007 groups participated in a previous study assessing the relationship between metabolic control of DM1 and nutritional status [16]. All data were extracted from the last visit recorded in the medical record for the study year. We also obtained the data 6–12 months before the diagnosis of diabetes diagnosis from the electronic medical record for the 2018 group. The Ethics Committee approved the data analysis, and the parents signed the informed consent. Patients diagnosed with DM1 who were not receiving insulin therapy, cases of neonatal diabetes, type 2 diabetes, and those with other diseases in addition to DM1 were excluded.

### Variables and data collection

The variables studied are presented in Table 1. Body mass index (BMI) was calculated using weight (SECA®700 scale, with a precision of 100 g) and height (SECA®220 stadiometer, with a precision of 1 mm). To describe nutritional status, the Z-scores for height and BMI were used, applying the LMS method [17, 18] and the World Health Organization (WHO) Child Growth Standards. The main study variable was the prevalence of overweight and obesity, which was determined according to the WHO references, as follows: for children under 5 years of age, overweight is defined as a BMI-Z > 2 SD and obesity as a BMI-Z > 3 SD, and for children over 5 years of age, overweight is defined as a BMI-Z > 1 SD and obesity as a BMI-Z > 2 SD.

**Table 1:** Characteristics of the children and adolescents studied

	*Normal weight (n* = *95)*	*Overweight and Obesity (n* = *41)*	*p*
*Age (years)*	*11.9* ± *2.8*^a^	*11.6* ± *3.1*^a^	*0.690*
*Age at diagnosis (years)*	*8 (4)*^*b*^	*7 (3.8)*^*b*^	*0.037*
*Children* < *5 years at diagnosis, n (%)*	*18 (18.9)*	*12 (29.3)*	*0.183*
*Children* > *10 years at diagnosis, n (%)*	*23 (24.2)*	*7 (17.1)*	*0.357*
*Children 5–10 years at diagnosis, n(%)*	*54 (56.8)*	*22 (53.7)*	*0.732*
*Sex (female), n (%)*	*42 (44.2)*	*21 (51.2)*	*0.452*
*Duration of DM1 (years)*	*3.1 (4.7)*^*b*^	*4.3 (5.6)*^*b*^	*0.163*
*Height Z-score*	*-0.38* ± *0.95*^*a*^	*0.08* ± *1.01*^*a*^	*0.013*
*HbA1c (%)*	*7.9 (1.6)*^*b*^	*7.7 (1.7)*^*b*^	*0.909*
*Insulin (UI/Kg/d)*	*0.73 (0.28)*^*b*^	*0.80 (0.29)*^*b*^	*0.566*
*Type of insulin, n (%)*			*0.009*
* - RAIA-LAIA*	*44 (46.3)*	*29 (70.7)*	
* - Regular-NPH*	*51 (53.7)*	*12 (29.3)*	
*Insulin pump, n (%)*	*3 (3.2)*	*4 (9.8)*	*0.198*
*Intensive therapy*^*c*^*, n (%)*	*56 (58.9)*	*36 (87.8)*	*0.007*
≥ *4 insulin doses, n (%)*	*36 (37.9)*	*27 (65.9)*	*0.003*
*Glucose monitoring, n (%)*			*0.588*
* - Test strips*	*67 (70.5)*	*27 (65.9)*	
* - CGM*	*28 (29.5)*	*14 (34.1)*	

^*a*^*mean* ± *SD;* ^*b*^*median (IQR);* ^*c*^ ≥ *3 insulin doses; RAIA-LAIA* rapid-acting and long-acting insulin analogs*, Regular-NPH* regular and NPH insulin*, CGM* continuous glucose monitoring

HbA1c, an important indicator of metabolic control in DM1, was determined in 1986 using chromatography (*Menarini*®) with JDS/JSCC calibration and in 2007 and 2018 using high-performance liquid chromatography (*Bio-Rad*®) with NGSP/DCCT calibration. To allow comparison, the values were expressed in NGSP/DCCT units (%) following the recommendations of the Global Consensus for Standardization of HbA1c measurement [19]. Intensive therapy was considered to be administration of three or more daily insulin injections or treatment with an external insulin pump, with dose adjustments based on at least four glucose readings per day with test strips or continuous glucose monitoring [20, 21].

### Statistical analysis

All statistical analyses were performed with SPSS Statistics, version 27.0 (IBM Corporation, Armonk, NY, USA). To determine the normality of the quantitative variables, the Lilliefors (Kolmogorov–Smirnov) test (*n* ≥ 50) or the Shapiro–Wilk test (*n* < 50) was applied. Qualitative variables were expressed as frequencies and percentages with their 95% confidence intervals (CI). Quantitative variables with a normal distribution were described using the mean and standard deviation (SD) and nonparametric variables with the median and interquartile range (IQR). The Chi^2^ test (Pearson), Fisher’s exact test, and comparison of proportions were used to compare the qualitative variables. The analysis of parametric quantitative variables between two groups was conducted using Student’s *t-*test and for nonparametric variables the Mann–Whitney U test. The ANOVA test was used for categorical variables with more than two groups with a normal distribution, and the Kruskal–Wallis test was used when the distribution was not normal. To test for differences within each group, a post hoc analysis was performed with the Bonferroni correction for multiple comparisons. To study the association between BMI-Z and the continuous quantitative variables, the Pearson test was used when the variables had a normal distribution or the Spearman test when they did not. To determine the risk of being overweight or obese, the odds ratio (OR) was calculated. A value of *p* < 0.05 was considered statistically significant.

## Results

**Fig. 1:**
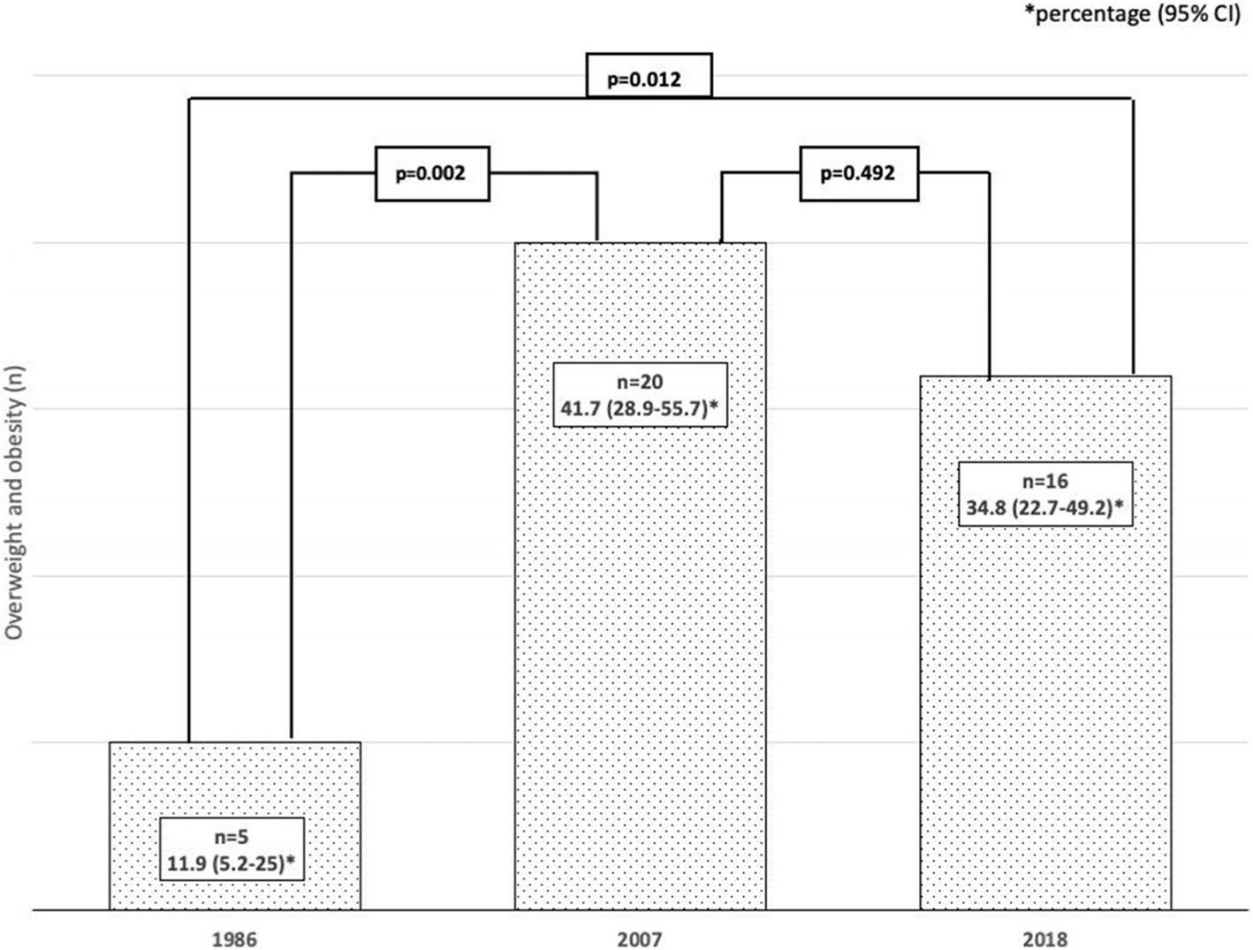
Prevalence of overweight and obesity in children and adolescents with type 1 diabetes

Table 1 describes the characteristics of the overweight and obese diabetic children and adolescents. Table 2 summarizes the prevalence of overweight and obesity in the three groups studied, and its evolution from 1986 to 2018 is depicted in Fig. 1. The proportion of overweight, including obesity, increased significantly from 1986 to 2007 and from 1986 to 2018, but not from 2007 to 2018.

**Table 2:** Prevalence of overweight and obesity in children and adolescents with type 1 diabetes

	*OVERWEIGHT*		*OBESITY*		*OVERWEIGHT* + *OBESITY*	
	*n*	*% (95% CI)*	*n*	*% (95% CI)*	*n*	*% (95% CI)*
*TOTAL (n* = *136)*	*35*	*25.7 (19.1–33.7)*	*6*	*4.4 (2–9.3)*	*41*	*30.2 (23.1–38.3)*
*1986 group (n* = *42)*	*5*	*11.9 (5.2–25.0)*	*0*	*-*	*5*	*11.9 (5.2–25.0)*
*2007 group (n* = *48)*	*16*	*33.3 (21.7–47.5)*	*4*	*8.3 (3.3–19.6)*	*20*	*41.7 (28.9–55.7)*
*2018 group (n* = *46)*	*14*	*30.4 (19.1–44.8)*	*2*	*4.4 (1.2–14.5)*	*16*	*34.8 (22.7–49.2)*

When comparing the overweight and obese diabetic children and adolescents with those of normal weight (Table 1), no significant differences were found in the distribution by age, sex, years with diabetes, continuous insulin infusion pump therapy, glucose monitoring, or proportion at diagnosis of children aged < 5 years and > 10 years.

No correlation was found between BMI-Z at the time of the study (median 0.42, IQR 1.1) and age at onset of diabetes (median 7.2, IQR 4.3) in the total sample (*p* = 0.202) or BMI-Z at diagnosis (mean 0.67 ± 1.14) and age at onset of diabetes (median 7.1, IQR 4.5) in the 2018 group (*p* = 0.632)*.*The age at diagnosis was lower in the overweight and obese children and adolescents (Table 1), but this significant difference was only observed in the 2018 group (*p* = 0.045). The children aged < 5 years had a BMI-Z at diagnosis similar to the children > 5 years (0.82 versus 0.62, *p* = 0.616) and at the time of the study (0.60 versus 0.45, *p* = 0.438), while the adolescents aged > 10 years had a significantly higher BMI-Z at diagnosis than those < 10 years (1.45 versus 0.45, *p* = 0.014) but not at the time of the study (0.51 versus 0.48, *p* = 0.878). Age at diagnosis < 5 years (OR = 1.77; 0.76–4.12) or > 10 years (OR = 0.65; 0.25–1.65) was not a risk factor for being overweight or obese. In the 2018 group, the rate of overweight and obesity was similar at diagnosis and at the end of the study (30.4% versus 34.8%, *p* = 0.092), and the risk estimate for the association between overweight and obesity at diagnosis and being overweight at the time of the study was not significant (OR = 0.214; 0.04–1.12).

**Fig. 2:**
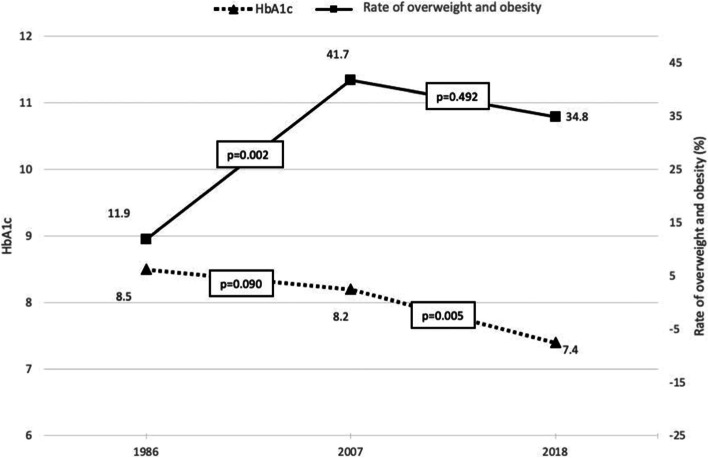
Metabolic control (HbA1c) and prevalence of overweight and obesity (%) in diabetic children and adolescents

**Fig. 3:**
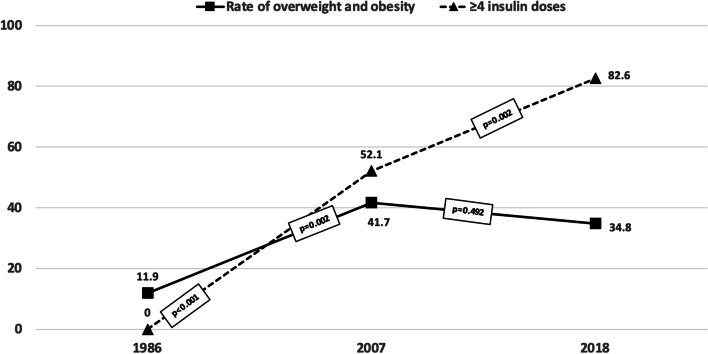
Administration of ≥ 4 daily insulin doses and prevalence of overweight and obesity (%) in diabetic children and adolescents

The amount of insulin used and metabolic control in the overweight and obese diabetic patients was similar to those with normal weight (Table 1). The insulin dose did not vary significantly from 1986 to 2018 (*p* = 0.493), but metabolic control improved from 1986 to 2018 (*p* < 0.001). The post-hoc analysis showed differences in HbA1c from 1986 to 2018 (8.5 to 7.4, *p* < 0.001) and from 2007 to 2018 (8.2 to 7.4, *p* = 0.005) but not from 1986 to 2007 (*p* = 0.090), the period in which the prevalence of overweight and obesity showed the greatest increase (Fig. 2). The use of intensive therapy (≥ 3 doses) was higher in the overweight and obese patients in all three groups (*p* = 0.007), but this type of therapy was only used in 2007 (91.8%) and 2018 (100%). In these patients, the use of ≥ 3 doses of insulin was not associated with a higher prevalence of overweight and obesity (*p* = 0.636). The proportion of patients with excess body mass receiving ≥ 4 daily insulin doses was also significantly higher (*p* = 0.003), but this type of therapy was only used in 2007 and 2018. From 2007 to 2018 this proportion rose from 52.1% to 82.6% (*p* = 0.002) but was not associated with changes in the rate of overweight and obesity in the period 2007 to 2018 (Table 1, Fig. 3).

**Fig. 4:**
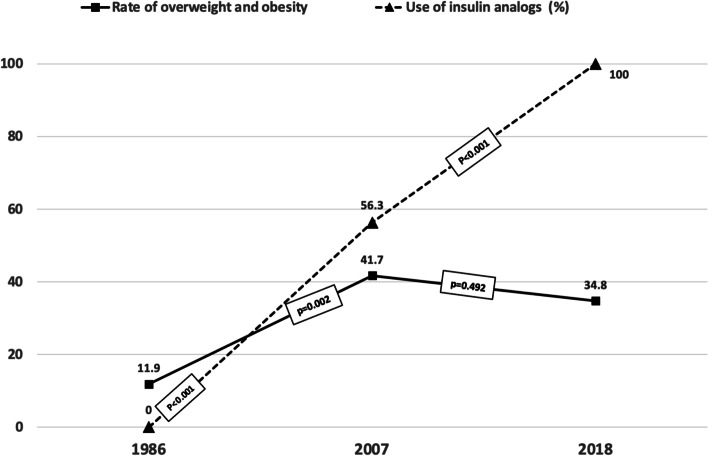
Use of insulin analogs and prevalence of overweight and obesity (%) in diabetic children and adolescents

The overweight and obese diabetic children and adolescents in the total sample showed a greater use of insulin analogs (*p* = 0.009). Rapid- and long-acting analogs were only used in 2007 and 2018 (56.3% to 100%, *p* < 0.001), but the rate of overweight and obesity showed no significant differences (*p* = 0.492) from 2007 to 2018 (Table 1, Fig. 4).

## Discussion

Over the three decades studied, diabetic children and adolescents showed improved metabolic control, and the use of rapid- and long-acting insulin analogs and intensive therapy with stricter glycemic monitoring has become widespread. In parallel, an increase in the prevalence of overweight and obesity in children and adolescents with DM1 of over 29% was observed from 1986 to 2007, with stabilization from 2007 to 2018, although with very high figures. These rates of overweight and obesity are similar to those reported in diabetic children in other Mediterranean areas [22].

In the sample studied, although the age at diagnosis of DM1 was significantly lower in the overweight and obese diabetic patients, there was no positive correlation between BMI-Z and age at diagnosis as observed by other authors [23], and the duration of diabetes showed no correlation with excess weight, as reported in other studies [24]. No significant differences were observed in BMI-Z at diagnosis in the children with DM1 who had onset before 5 years of age, as postulated by the accelerator hypothesis, and being younger than 5 years of age at diagnosis was not associated with increased risk of overweight or obesity [9]. The patients with onset from the age of 10 years had higher BMI-Z at diagnosis, but being > 10 years of age at diabetes onset was not associated with an increased risk of overweight or obesity.

Intensive therapy and improved metabolic control have been associated with excessive weight gain in diabetic children and adolescents [11, 25] which could partially diminish the beneficial effects of improved diabetes control on microvascular and macrovascular complications [26], although other studies have not been able to confirm this relationship [22]. In our study, metabolic control improved throughout the period analyzed (Fig. 2), and the use of intensive therapy and insulin analogs in the 2007 and 2018 groups was not associated with a higher risk of overweight and obesity. The number of patients on intensive therapy and using insulin analogs increased significantly from 1986 to 2007 as did the rate of overweight and obesity, coinciding with the obesity pandemic. From 2007 to 2018 the proportion of children and adolescents receiving higher doses of insulin and using insulin analogs continued to increase to 100% in 2018, but the prevalence of overweight and obesity observed did not change from 2007 to 2018 (Figs. 3 and 4), in contrast to other studies [27].

**Fig. 5:**
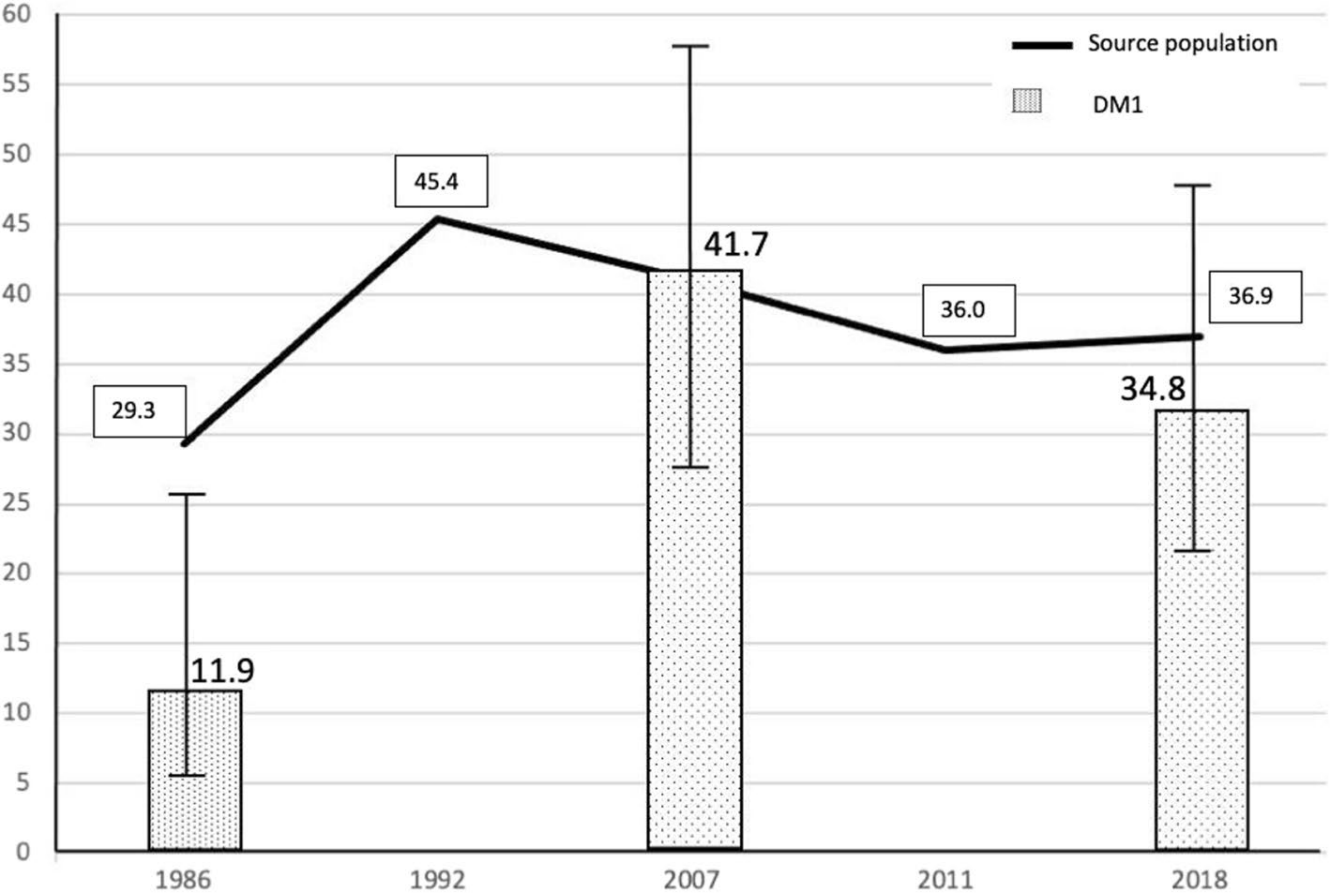
Percentage (error bars show 95% CI) of overweight and obesity in the children and adolescents with type 1 diabetes studied and prevalence of overweight and obesity (%) in children and adolescents in the source population (2)

The various environmental and biological factors that contribute to overweight and obesity in the general population, other than the insulin regimen, could partly explain the evolution of the prevalence of overweight and obesity in diabetic children and adolescents during the period studied [8, 28]. The global rise in childhood overweight and obesity observed in the last forty years has shown a tendency to stabilize in recent studies [3]. The pediatric population in our healthcare area, which was the source of the sample studied, has shown a similar evolution [2] with very high figures for overweight and obesity. Nonetheless, as other authors have reported [10], the incidence of DM1 does not show a linear relationship with excess body mass, and during the period studied, the incidence of DM1 in children and adolescents continued to increase steadily in Europe [4], North America [29, 30], and Spain [5]. As other authors have observed in the adult population with DM1 [31], our findings reveal an increase in the prevalence of overweight and obesity in diabetic children and adolescents, with stabilization in the last ten years, similar to that seen in the population from which the sample was drawn [2, 32], as shown in Fig. 5. The relationship between the distribution of overweight and obesity in diabetic children and adolescents and the childhood obesity epidemic has also been suggested by other authors [28]. Factors such as genetic predisposition [33], an obesogenic environment [24], and the microbiome [8], which promote obesity and increased insulin resistance in the general population, would also affect diabetic children and adolescents.

Although the rate of obesity and overweight in diabetic children and adolescents has stabilized in our healthcare area, the figures are still very high, contributing to an increased risk of hypertension, dyslipidemia, and early cardiovascular disease in this susceptible population [30]. The effect of overweight and obesity on the metabolic syndrome in diabetic patients has been studied in children [13, 30] and especially in adults [11, 31], in whom obesity has been associated with an increased risk of hospitalization [34]. These findings underscore the importance of early diagnosis and treatment of excess weight and its metabolic consequences in this at-risk group. Diabetes management encouraging healthy eating and exercise is key to preventing obesity, overweight, and cardiovascular disease.

## Limitations

This study has several limitations, including its retrospective design and that it was undertaken at a single center serving a single health department, and therefore the number of patients was small. Other limitations were the lack of data on the pubertal status of the patients and the lack of waist circumference to estimate the metabolic syndrome with the criteria currently used. As strengths, this study covers the evolution of three groups of children with DM1 (*n* = 136) over the last 30 years in a health area for which we have data on overweight and obesity in the source population. The criteria used to define these groups were homogeneous and based on international standards.

## Conclusions

The number of overweight and obese diabetic children and adolescents has stabilized in the last decade but continues to be very high. In each group, the prevalence mirrored that observed in the source population. If, as our study suggests, the increased prevalence of overweight and obesity in some diabetic patients could simply reflect an increase in obesity in the general population, continued identification of the emerging factors behind the obesity epidemic in the general and diabetic populations will be needed in order to implement health policies that promote healthy eating and reduce sedentary lifestyles. The relationship between overweight and obesity in diabetic children with age at diagnosis, accelerated onset, intensive therapy, duration of diabetes, and its long-term effects requires further investigation.

## Data Availability

The datasets used and/or analyzed during the current study are available from the corresponding author on reasonable request.

## Abbreviations

DM1: Type 1 diabetes
WHO: World Health Organization
BMI: Body mass index
BMI-Z: Body mass index z-score
CI: 95% Confidence interval
SD: Standard deviation
IQR: Interquartile range
OR: Odds ratio
RAIA-LAIA: Rapid-acting and long-acting insulin analogs
Regular-NPH: Regular and NPH insulin
CGM: Continuous glucose monitoring
